# Alcohol Drinking Patterns and Differences in Alcohol-Related Harm: A Population-Based Study of the United States

**DOI:** 10.1155/2014/853410

**Published:** 2014-06-25

**Authors:** D. Antai, G. B. Lopez, J. Antai, D. S. Anthony

**Affiliations:** ^1^City University London, School of Health Sciences, Centre for Public Health Research, Northampton Square, London EC1V 0HB, UK; ^2^Division of Global Health & Inequalities, The Angels Trust, Abuja, Nigeria; ^3^Department of Psychology, Queens College, The City University of New York, Long Island City, NY 11101, USA

## Abstract

Alcohol use and associated alcohol-related harm (ARH) are a prevalent and important public health problem, with alcohol representing about 4% of the global burden of disease. A discussion of ARH secondary to alcohol consumption necessitates a consideration of the amount of alcohol consumed and the drinking pattern. This study examined the association between alcohol drinking patterns and self-reported ARH. Pearson chi-square test (*χ* ^2^) and logistic regression analyses were used on data from the National Comorbidity Survey Replication (NCS-R). The NCS-R is a cross-sectional nationally representative sample. Data was obtained by face-to-face interviews from 9282 adults aged ≥18 years in the full sample, and 5,692 respondents in a subsample of the full sample. Results presented as odds ratio (OR) and 95% confidence intervals (95% CI). Alcohol drinking patterns (frequency of drinking, and drinks per occasion) were associated with increased risks of self-reported ARH; binge or “risky” drinking was strongly predictive of ARH than other categories of drinks per occasion or frequency of drinking; and men had significantly higher likelihood of ARH in relation to frequency of drinking and drinks per occasion. Findings provide evidence for public health practitioners to target alcohol prevention strategies at the entire population of drinkers.

## 1. Introduction

Alcohol use and associated alcohol-related harm (ARH) are among the most prevalent and important public health problems plaguing this generation [1]. Alcohol represents about 4% of the global burden of disease [2]; this burden is higher in high-income countries and among men [3]. Consequently, the global public health burden and economic costs of alcohol use are high [4]. Alcohol consumption can result in several negative consequences, ranging from health to social consequences and affecting friends or family and the workplace. When discussing ARH following alcohol consumption, whether the effect is on the general health [5, 6] or on areas such as work, social relations, and economy [7, 8], it is pertinent to consider the amount of alcohol consumed as well as the drinking pattern. For example, recurrent heavy episodic drinking (HED) equivalent to ≥5 drinks (also known as binge or “risky” drinking) [9] is a drinking pattern that appears to exacerbate population ARH from alcohol consumption; this alcohol consumption pattern has been shown to be a problem not only in the US [10], but in other regions of the world such as the Nordic region [11] and the United Kingdom [12]. The value of also considering the frequency of consumption without overrelying on quantity measures has been emphasized by such authors as Fillmore and Jude [13].

Recommendations on maximum daily alcohol intake vary both between and within countries [14]. Based on lifetime risk of alcohol-attributable mortality [15], it is recommended that the daily intake of alcohol for both men and women should not exceed two drinks, with three or four drinks considered as tolerable for occasional drinking. In the USA, the National Institute on Alcohol Abuse and Alcoholism (NIAAA) defines binge drinking for a typical adult as an alcohol drinking pattern that brings the blood alcohol concentration to ≥0.08 g per cent (equivalent to ≥5 drinks for males or ≥4 drinks for females, in ~2 h) [16]. A standard US “drink” in the above-mentioned definition refers to half an ounce of alcohol (i.e., 14 g ethanol) (or 8 g in the United Kingdom). Thus, one standard US drink is contained in one 12-oz. bottle or can of typical (5% alcohol by volume [ABV]) beer; one 5-oz. glass of typical (12% ABV) wine; or one 1.5-oz. shot of (40% ABV) distilled spirits or liquor (e.g., gin, rum, vodka, and whiskey).

The main focus in surveys of ARH has historically been on characteristics and behavior of respondents, combined with sample designs in which one respondent is chosen per household to minimize cross contamination. Little attention has often been paid to social interactions (i.e., an individual's drinking behavior that is considered as problematic and a reaction by someone other than the drinker) and contexts. There is however little agreement about methods of measuring drinking-related social harm in population surveys in spite of the growing consensus about how to measure patterns and amounts of drinking [17]. Epidemiological research on ARH has often followed two main traditions: clinical and socioepidemiological [18]. The clinical or “characteristics of the individual” approach, which is rooted on the notion of diseases as discrete entities, views problems associated with excessive alcohol intake as part of a condition with a characteristic natural history and is measured using aggregate measures of alcohol problems which operationally define a clinical entity. The socioepidemiological or “problems” approach, which stresses the importance of the interaction between the individual and the social environment in the appearance of ARH, explores results with an aggregate measure or score composed of all or some of the items of alcohol problems [19]. These measures can be considered as indices of a number of problems all having the same value, with ARH determined by arbitrary cut-off points. Various analyses use individual-level measures or items [20], such as those from the Alcohol Use Disorders Identification Test (AUDIT) developed by the World Health Organization (WHO) to screen for people at risk of developing alcohol problems [21]. Other options for assessing survey data on ARH beyond the individual level include samples and questions designed to obtain information from both sides of an interaction (i.e. an individual's drinking behavior that is considered as problematic and a reaction by someone other than the drinker) [22] and analyzing survey data in conjunction with aggregate statistics or with respondents being clustered to aggregate their responses [23]. These analytical options are beyond the scope of the present paper; the present study analyses individual items because ARH in the National Comorbidity Survey Replication (NCS-R) is measured as single items.

Potentially influential predictors of progression to alcohol-related harm besides alcohol consumption include socioeconomic position (SEP) [24, 25], early age of alcohol initiation [26], being male (although females appear to suffer serious negative consequences of alcohol consumption earlier and to a greater degree than men) [27, 28], family history [29], comorbid substance use, with alcohol use increasing the risk for other drug use disorders [30, 31], poor physical and mental health [32], sex differences [33, 34], and ethnicity [35]. The adverse effects of alcohol drinking behavior affect not only the index drinker but also family members of the drinker [36] and the society as well.

There is an increasing shift in paradigm from mean alcohol consumption as a significant determinant of ARH at the individual and population level to drinking patterns [37], given that mean alcohol consumption is an incomplete predictor of risk. This study is unique in specifically assessing ARH affecting the whole family and/or as a social problem with work, responsibilities, and others. This association is important from a public health perspective, as prevention programs could potentially limit the development of later adverse outcomes, thus reducing individual pain and suffering and preventing socioeconomic and healthcare-related costs to the society.

## 2. Hypotheses

The following hypotheses were examined.

*Hypothesis I*. Alcohol drinking patterns (i.e., frequency of drinking and number of drinks per occasion) will increase the risk of ARH, even after controlling potential confounders.

*Hypothesis II*. Drinks per occasion will be more predictive of ARH than frequency of drinking. 

This study therefore aimed to (i) describe the prevalence of two alcohol drinking patterns and self-reported ARH in the study sample; (ii) examine whether alcohol drinking patterns increased the risk of self-reported ARH; (iii) examine whether drinks per occasion will be more predictive of ARH; and (iv) assess the correlates of ARH among respondents.

## 3. Methods

### 3.1. Study Design

Data was obtained from the National Comorbidity Survey Replication (NCS-R) conducted between February 2001 and April 2003. The NCS-R is a cross-sectional nationally representative sample of English-speaking adults aged ≥18 years in the noninstitutionalized civilian population of the 48 coterminous states in the US. Interviews were conducted face to face in the homes of respondents. Detailed descriptions of the methodology, weighting, and sampling procedures used in the NCS-R have been previously provided elsewhere [38]. Briefly, the NCS-R interviews consisted of two parts administered in one session. Part I was administered to the full sample of 9282 respondents and included a demographic section that assessed sex, age, education, marital status, and current household income. Part II assessed chronic physical disorders, risk factors, and costs of illness and was administered to a probability subsample of 5,692 respondents of Part I using Version 3.0 of the World Health Organization (WHO) Composite International Diagnostic Interview (CIDI) [38, 39], a structured diagnostic interview designed to generate diagnoses of commonly occurring mental disorders according to the definitions and criteria of both the ICD-10 [40] and* DSM-IV* Axis I [41] systems. The alcohol module was administered to all respondents in the Part II sample.

### 3.2. Ethical Considerations

Approval for these recruitment and consent procedures were obtained from the Human Subjects Committees of Harvard Medical School and the University of Michigan. The response rate was 70.9%.

### 3.3. Study Outcomes

Alcohol-related harm: five measures of ARH in the preceding 12 months were assessed: (1) drinking problem causing family/friend argues/problems; (2) drinking ever interfered with work/school/job/home; (3) family worries or complains about alcohol use; (4) alcohol use causing problems/argue with others; and (5) alcohol interfered with responsibilities. The analysis of ARH is kept at the item level (rather than exploring results with a score composed of all or some of the items), because in the National Comorbidity Survey Replication (NCS-R) alcohol-related harm (social harm) is measured as single items.

### 3.4. Study Exposures

Alcohol drinking patterns examined include (1) frequency of drinking at least 1 drink in the past 12 months in the past year (daily, 3-4 days per week, 1-2 days per week, 1–3 days per month, less than once a month, and did not drink in past 12 months); and (2) number of drinks per day each time you drank (≤2 drinks per occasion, 2–4 drinks, and ≥5 drinks or binge drinking).

The following predictors were identified* a priori* as having potential confounding influence on the relationship between alcohol consumption and progression to the development of ARH, including sociodemographic characteristics such as sex, men are more likely to drink, consume more alcohol, and have alcohol use disorders than women [30]; age of cohort, younger cohorts also showed higher risk of alcohol dependence and current alcohol abuse [42]; employment status; household income; education level, individuals of low socioeconomic status suffer more harmful consequences of drinking than their counterpart in higher socioeconomic positions [24, 25]; ethnicity, consequences of alcohol consumption and trajectories of alcohol problem are more profound in some ethnic groups than others [28, 43]; physical and mental health ratings, poor physical and mental health is often associated with alcohol use disorders [32]; and smoking, studies have shown a strong tendency for cigarette smoking and alcohol dependence to cooccur and both are associated with other drug use disorders [44, 45].

### 3.5. Statistical Analysis

Pearson chi-square test (*χ* ^2^) was used to assess statistical significance of differences in the frequency and distribution of ARH and alcohol drinking patterns among respondents. Statistical significance was set at *P* < 0.05, and all *P* values are two sided. Logistic regression models were used to examine the association between alcohol drinking patterns and ARH, by applying different multivariate models as suggested by Greenland and Lash [46]. Model 1 was the crude association between the alcohol drinking pattern and the different forms of ARH to calculate the unadjusted effect of the alcohol drinking pattern. Only variables found to be significant in bivariate models and fulfilling the criteria for being confounders were included in multivariate analyses [46]. Predictors, selected based on good theoretical reasons for inclusion of predictors, were adjusted for in Model 2 by being added into the model simultaneously since our goal was theory testing [47]. We also did a series of sensitivity analyses in which drinks per occasion and frequency of drinking were both included as exposure variables in each model with ARH and the other covariates. This was intended to further tease apart the independent effects of frequency and quantity of alcohol; this could account for the effects of individuals who drink a lot per occasion amongst those who drink alcohol frequently, as well as the effects of those who consume modest amounts of alcohol per occasion amongst frequent drinkers. Results were presented as odds ratio (OR) and 95% confidence intervals (95% CI). Additional bivariate analyses were conducted between education (years), frequency of drinking, drinks per occasion, and alcohol-related harm. Statistical analyses were carried out using the Statistical Package for the Social Sciences (SPSS).

## 4. Results 

### 4.1. Prevalence and Frequency of Alcohol-Related Harm by Alcohol Drinking Patterns and Sociodemographic Characteristics of Respondents

The most common types of alcohol-related harm were “family worries or complains about alcohol use” (12%), “drinking problem causing family/friend argues/problems” (7%), and “drinking interfered with work/school/job/home” (5%). The least common at 1% were “alcohol use causing problems with others” (1%) and “alcohol interfered with responsibilities,” respectively. The differences in the distribution of all the measures of ARH and alcohol drinking patterns among respondents were statistically significant for the overall sample and across respondents' age and ethnicity. Individuals who did not drink (i.e., abstainers) more frequently reported almost all the measures of ARH compared to those who drank daily; this proportion was however not higher than that of light consumers (referred to in this study as those who drank less than once/month and 1–3 days/month). In contrast, individuals who drank the least amount of alcohol per occasion (i.e., ≤2 drinks/occasion) reported a higher prevalence of ARH compared to light consumers (referred to in this study as those who drank 2–4 drinks/occasion). Males were more frequently associated with several measures of ARH (family worried or complains about alcohol use; alcohol use caused problems with others; and alcohol interfered with responsibilities). The prevalence of all the measures of ARH was significantly higher amongst individuals aged 49 years or younger and non-Hispanic blacks. Individuals with higher education (≥16 years) reported the least prevalence of ARH (family worries or complains about alcohol use) (Table 1).

### 4.2. Association between Alcohol Drinking Patterns, Sociodemographic Characteristics, and Alcohol-Related Harm

The association between frequency of drinking and ARH is presented in Table 2. Crude analysis (Model 1) showed strong significant and positive associations between more frequent drinkers (daily and 3-4 days/week) and several ARH (family worries or complains about alcohol use; alcohol use causing problems with others; and alcohol interfered with responsibilities) compared to abstainers, that is, those who did not drink. In relation to the other two measures of ARH (drinking ever interfered with work/school/job/home and drinking problem causing family/friend argues/problems), the association was significantly negative among less frequent drinkers (1-2 day/week, 1–3 days/month, and less than once/month) compared to abstainers. After adjusting for confounders (Model 2), the associations were attenuated, whilst remaining largely significant.

In Table 3, the crude analyses (Model 1) in the association between number of drinks per occasion and ARH showed increasing and significantly higher risks of all the measures of ARH in relation to higher frequency of drinks per occasion (2–4 drinks/occasion and ≥5 drinks/occasion) compared to those who had ≤2 drinks/occasion. Adjusting for confounders (Model 2) only slightly attenuated these associations. The odds of reporting ARH were higher with increasing number of drinks per occasion compared to just having ≤2 drinks/occasion, with binge or “risky” drinking (i.e., ≥5 drinks per occasion) being consistently associated with higher odds in relation to all the five measures of ARH. The odds ratio (ORs) associated with drinks per occasion were generally higher than those associated with frequency of drinking.

### 4.3. Sociodemographic Factors Associated with Alcohol-Related Harm

In the multivariate analyses, male drinkers had significantly higher odds of ARH compared to female drinkers when alcohol consumption was measured either as frequency of drinking or as drinks per occasion. Younger individuals were generally at higher odds of ARH compared to those aged ≥65 years; the strongest odds ratio (ORs) was found among individuals aged ≤34 years and frequency of drinking (OR = 7.20) and number of drinks per occasion (OR = 5.46). The odds of ARH (drinking problem causing family/friend argues/problems) in association with frequency of drinking and number of drinks per occasion were higher among individuals with lower educational level compared to those with higher education (≥16 years). Income was only associated with ARH (family worries or complains about alcohol use) in relation to both alcohol drinking patterns, with higher risks found among individuals with lower income. The odds of ARH associated with both drinking patterns were generally lower among non-Hispanic blacks and Hispanic/others compared to non-Hispanic whites, with the exception of “alcohol interfered with responsibilities” in relation to frequency of drinking for which non-Hispanic blacks had 55% higher odds (OR = 1.55) than non-Hispanic whites. The odds of ARH (“family worries or complains about alcohol use”) in relation to frequency of drinking were higher for those who reported good (OR = 1.51) and fair/poor (OR = 1.45) physical health compared to those who reported very good/excellent physical health. Individuals reporting fair/poor mental health had higher odds of ARH (“family worries or complains about alcohol use” and “alcohol use causing problems with others”) in relation to both alcohol consumption measures.

### 4.4. Sensitivity Analyses

Sensitivity analyses with daily drinking amounts and frequency both included with the other covariates resulted in the association between the drinking patterns and two measures of ARH (drinking ever interfered with work/school/job/home and drinking problem causing family/friend argues/problems) and the relation between drinks per occasion and the ARH “measure alcohol interfered with responsibilities” becoming statistically nonsignificant. There was a positive association between the frequency of drinking category “daily” and three ARH measures (“family worries or complains about alcohol use,” “alcohol use causing problems with others,” and “alcohol interfered with responsibilities”) compared to their counterparts who did not drink; all other categories of frequency of drinking were negatively associated with these three ARH measures. Respondents in the drinks per occasion category “≥5 drinks/occasion” had an almost threefold higher risk of having “family worries or complains about alcohol use” compared to those who drank ≤2 drinks/occasion.

## 5. Discussion

This study examined the prevalence of ARH and the association between alcohol use patterns self-reported ARH and yielded 4 key findings. First, prevalence of ARH ranged between 1% (“alcohol use causing problems with others” and “alcohol interfered with responsibilities”) and 12% (“family worries or complains about alcohol use” and that abstainers and those with the least amount of drinking per occasion exhibited higher prevalence of alcohol-related harm than light consumers, irrespective of the alcohol drinking pattern). The prevalence of ARH found in the present study is within the range of those reported in the US (5.3%) [48], Spanish (6.5%) [49], and Swiss (7.7%) studies [50], although these figures might not be directly comparable due to definitions of alcohol-related harm used in these studies. The indication that individuals with the least amount of alcohol drunk per occasion exhibited higher proportions of ARH than light consumers contributes to the empirical evidence that a major part of the total burden of ARH is contributed by moderate drinkers—the “prevention paradox”—and this states that “the bigger part of ARH may originate among moderate drinkers, even though the risk of negative consequences is by far the highest among subjects with high-risk drinking.” Although ARH was largely attributed to the high proportion of low to moderate drinkers who constituted to the vast majority of drinkers in the study population and is in line with findings from other studies [51, 52], this may imply that public health prevention strategies aimed at the entire population of drinkers may be more effective for most ARH than strategies aimed only at the smaller subgroup of high-risk drinkers in the population as previously suggested [8]. However, results of the logistic regression models indicating that risky drinkers had higher odds ratio of ARH would favor targeting high-risk drinkers.

Second, alcohol drinking patterns were associated with increased risks of self-reported ARH; this association was largely dose-dependent, corroborating findings from previous studies indicating that positive relations between overall intake established and patterns of drinking, especially irregular heavy drinking [53], are related to nonmedical consequences of drinking. Although a few medical conditions, such as cardiovascular disease, show a protective effect of moderate consumption of alcohol, most conditions revealed a positive linear or exponential relationship with the volume of alcohol consumption [54]. In addition, we found support for our first hypothesis that alcohol drinking patterns will increase the risk of alcohol-related harm, even after controlling for potential confounders. It was not possible to determine whether abstainers reporting ARH recently quit drinking due to these problems; this may be due to the cross-sectional nature of the data, which precludes the drawing of causal inference.

Third, binge or “risky” drinking was strongly predictive of ARH than other categories of drinks per occasion or frequency of drinking. This finding is consistent with those from other studies among a sample of US adults [7], high school students [55], and a Swedish population attending primary health care [11], thus providing evidence for our second hypothesis that drinks per occasion (especially binge or “risky” drinking) will be more predictive of ARH than frequency of drinking. Despite binge or “risky” drinking being well known as harmful to health, it is however possible that “risky” drinking through its association with other health-risk behaviors, such as smoking [7], plays a role in causing alcohol-related harm. Smoking was however not significantly associated with alcohol drinking patterns and alcohol-related harm in this study. Comprehensive alcohol prevention strategies should also include efforts to reach high consumers.

Other noteworthy findings include male drinkers being more likely than female drinkers to report ARH when alcohol consumption was measured either as frequency of drinking or as drinks per occasion. Similar findings have been reported in India [56]. Plausible explanation, besides biological differences in alcohol metabolism between men and women, may be the higher exposure opportunities among men due to psychological, family, and social factors [57], given that alcohol use and related harm are linked to gender roles, expectations, and culture in society [58]. Evidence on the sex differences found in this study echoes the importance of identifying the differential effects of alcohol on men and women. Individuals with lower educational attainment and income were more likely than those of higher education and higher income, respectively, to report ARH in relation to both alcohol drinking patterns, which is consistent with findings from other studies [26–29]. These findings correspond to social gradients in health, in which those with lower socioeconomic circumstances have worse health [59] and may be a result of, and a way of coping with, harsh economic and social conditions.

Non-Hispanic blacks and Hispanic/other ethnic groups were generally more less likely to report ARH associated with both drinking patterns compared to non-Hispanic whites, with the exception of “alcohol interfered with responsibilities” in relation to frequency of drinking which non-Hispanic blacks were more likely to report than non-Hispanic whites. These mixed findings may be linked with social and cultural factors such as ethnic groups' norms and attitudes regarding alcohol use and biological factors [59–61]. Acculturation, the partial or complete adoption of the original drinking pattern of an ethnic group resembling that of the overall population, may also contribute to minority drinking patterns; this in turn may be influenced by country of origin, religious beliefs, family traditions, and so forth [62]. Non-Hispanic blacks and Hispanics/others have been reported to exhibit more conservative alcohol norms and attitudes than non-Hispanic whites [63], which is reflected in the greater abstention rates and lower likelihood of ARH among these ethnic groups compared to non-Hispanic whites, although consequences of alcohol consumption and trajectories of ARH are more significant in some ethnic groups than others [35]. These findings call for a general awareness of ethnic differences that what can assist public health professionals in identifying the subpopulations most at risk for developing particular alcohol-related problems so as to appropriately target prevention strategies [64].

Finally, we found higher odds of ARH among those who reported good and fair/poor physical and mental health compared to those who reported very good/excellent physical health, which is consistent with findings from a California study [65], in which poor physical and mental health was associated with binge drinking and adverse experiences among women, and a cross-sectional study providing evidence of positive association of risk drinking with harm, including impaired mental and physical health, cognitive ability, and ability to perform activities of daily living [66]. Identifying individuals who drink and have poor physical and mental health is a key step in comprehensive prevention and intervention efforts that address physical and mental health symptoms.

By including frequency of drinking and drinks per occasion with the other covariates in the sensitivity analysis accounting for the distribution of those who drink frequently among those who consume much alcohol per occasion, and vice versa, the odds of “family worries or complains about alcohol use” increase almost threefold when the amount of alcohol consumed was ≥5 drinks/occasion. This finding provides added support for our earlier finding that binge or “risky” drinking was strongly predictive of ARH than other categories of drinks per occasion or frequency of drinking.

### 5.1. Strengths and Limitations

Among the limitations of this study are that all ARHs are based on self-report and thereby subject to recall error with the risk of underestimation of true levels of drinking. The association between drinking patterns and social harm relies on the subjective causal relationship of the reported ARH to alcohol; however, the cross-sectional design precludes inferences as to causality and longitudinal studies are needed to confirm the present findings and examine mechanisms underlying the predictors of the outcomes. We did not provide information on the severity or frequency of ARH since we only used binary variables to assess ARH; this may underestimate the share of the burden of total harm that could potentially be attributed to heavy drinkers/frequent binge drinkers. We emphasize that possible information bias of exposure could lead to nondifferential misclassification since it does not depend on the outcome; this could potentially result in underestimation of the association of interest [46]. There might also be underreporting in self-reported data of alcohol use. Strengths of the current study include the importance of the key findings to alcohol prevention strategies, the nationally representativeness of the sample, and the incorporation of comprehensive control of potential confounders (i.e., age, education, income, place of residence, ethnicity, physical and mental health status, and smoking).

### 5.2. Policy Implications

The strategic approaches to tackling ARH differ markedly across countries. This study provides evidence to support important tasks needed to prevent ARH and suggests that comprehensive alcohol prevention strategies within countries should also include making strong commitments is public health prevention strategies aimed at the entire population of drinkers; this may be more effective for most alcohol-related problems and preferred to strategies aimed only at the smaller subgroup of high-risk drinkers in the population. However, efforts should also be made to reach high consumers.

## 6. Conclusions 

This study provides evidence that alcohol drinking patterns were associated with increased risks of self-reported ARH and that drinks per occasion especially binge or “risky” drinking was strongly predictive of ARH than other categories of drinks per occasion or frequency of drinking. Males had significantly higher likelihood of ARH in relation to frequency of drinking and drinks per occasion. There is a need for comprehensive public health alcohol prevention strategies at the entire population of drinkers, including efforts aimed at the smaller subgroup of high-risk drinkers.

## Figures and Tables

**Table 1:** Frequency of alcohol-related harm by alcohol drinking patterns and sociodemographic characteristics of respondents.

	Family worries or complains about alcohol use		Alcohol use causing problems with others		Alcohol interfered with responsibilities		Drinking ever interfered with work/school/job/home		Drinking problem causing family/friend argues/problems	
	No	Yes	No	Yes	No	Yes	No	Yes	No	Yes
	*N* (%)	*N* (%)	*N* (%)	*N* (%)	*N* (%)	*N* (%)	*N* (%)	*N* (%)	*N* (%)	*N* (%)
Frequency of drinking	*P* < .001		*P* < .001		*P* < .001		*P* < .001		*P* < .001	
Daily	320 (16)	478 (6)	17 (12)	461 (6)	20 (15)	441 (6)	154 (16)	202 (18)	219 (18)	137 (15)
3-4 days/week	284 (14)	560 (7)	26 (18)	534 (6)	20 (15)	514 (6)	144 (15)	182 (16)	183 (15)	142 (16)
1-2 day/week	350 (18)	1461 (18)	30 (21)	1432 (18)	23 (17)	1409 (18)	157 (16)	238 (21)	223 (18)	173 (20)
1–3 days/month	257 (13)	1709 (21)	24 (16)	1686 (21)	19 (14)	1667 (21)	123 (12)	145 (13)	136 (11)	133 (15)
Less than once/month	327 (17)	2692 (32)	24 (16)	2669 (33)	33 (25)	2636 (33)	160 (16)	162 (14)	169 (14)	152 (17)
Did not drink (abstainers)	431 (22)	1306 (16)	25 (17)	1280 (16)	17 (12)	1263 (16)	248 (25)	199 (18)	300 (24)	146 (17)
Total	**1969**	**8206**	**146**	**8062**	**132**	**7903**	**986**	**1128**	**1230**	**883**
Drinks per occasion	*P* < .001		*P* < .001		*P* < .001		*P* < .001		*P* < .001	
≤2 drinks/occasion	2570 (61)	423 (35)	36 (38)	2535 (62)	30 (37)	2505 (63)	167 (29)	289 (38)	218 (29)	238 (41)
2–4 drinks/occasion	1000 (24)	329 (28)	32 (34)	969 (24)	29 (36)	940 (23)	162 (29)	223 (30)	210 (28)	174 (30)
≥5 drinks/occasion	609 (15)	439 (37)	27 (28)	582 (14)	22 (27)	560 (14)	238 (42)	244 (32)	318 (43)	166 (29)
Total	**4179**	**1191**	**95**	**4086**	**81**	**4005**	**567**	**756**	**746 **	**578**
Sex	*P* < .001		*P* < .001		*P* < .001		*P* = .430		*P* = .206	
Male	6980 (40)	1491 (64)	6864 (40)	117 (68)	6766 (39)	100 (63)	731 (65)	634 (64)	562 (63)	804 (65)
Female	10513 (60)	831 (36)	10458 (60)	55 (32)	10397 (61)	60 (37)	400 (35)	354 (36)	324 (37)	428 (35)
Total	**17493**	**2322**	**17322**	**172**	**17163**	**160**	**1131**	**988**	**886**	**1232**
Age (group)	*P* < .001		*P* = .020		*P* < .001		*P* = .003		*P* < .001	
≤34 years	6157 (35)	780 (34)	6086 (35)	71 (41)	6002 (35)	84 (53)	396 (35)	362 (36)	354 (40)	404 (33)
35–49 years	5443 (31)	923 (40)	5386 (31)	57 (33)	5332 (31)	55 (34)	429 (38)	400 (41)	316 (35)	512 (41)
50–64 years	3463 (20)	469 (20)	3430 (20)	33 (19)	3415 (20)	15 (9)	217 (19)	186 (19)	149 (17)	254 (21)
≥65 years	2430 (14)	150 (6)	2420 (14)	11 (7)	2414 (14)	6 (4)	89 (8)	40 (4)	67 (8)	62 (5)
Total	**17493**	**2322**	**17322**	**172**	**17163**	**160**	**1131**	**988**	**886**	**1232**
Education (years)	*P* < .001		*P* = .074		*P* = .173		*P* = .968		*P* = .104	
0–11	3388 (19)	621 (27)	3343 (19)	45 (26)	3318 (19)	25 (16)	300 (26)	266 (27)	226 (26)	340 (28)
12	5136 (29)	719 (31)	5085 (29)	49 (28)	5047 (29)	39 (24)	333 (29)	295 (30)	261 (29)	367 (30)
13–15	4620 (26)	627 (27)	4574 (26)	46 (27)	4523 (27)	51 (32)	313 (29)	273 (28)	238 (27)	349 (28)
≥16	4349 (26)	355 (15)	4320 (26)	32 (19)	4275 (25)	45 (28)	185 (16)	154 (15)	161 (18)	176 (14)
Total	**17493**	**2322**	**17322**	**172**	**17163**	**160**	**1131**	**988**	**886**	**1232**
Income	*P* < .001		*P* = .600		*P* = .688		*P* = .293		*P* = .498	
<$20,000	4830 (28)	756 (33)	4781 (28)	48 (28)	4735 (28)	46 (29)	374 (33)	290 (29)	274 (31)	390 (32)
$20,000–29,999	2692 (15)	325 (14)	2659 (15)	30 (17)	2644 (15)	19 (12)	145 (13)	136 (14)	124 (14)	157 (13)
$30,000–49,999	3738 (21)	492 (21)	3703 (21)	37 (22)	3665 (21)	36 (22)	229 (20)	218 (22)	198 (22)	251 (20)
≥$50,000	6233 (36)	749 (32)	6179 (36)	57 (33)	6119 (36)	59 (37)	383 (34)	344 (35)	290 (33)	434 (35)
Total	**17491**	**2322**	**17322**	**172**	**17163**	**160**	**1131**	**988**	**886**	**1232**
Ethnicity	*P* < .001		*P* = .005		*P* = .002		*P* = .011		*P* = .024	
Non-Hispanic black	6519 (37)	1043 (45)	6441 (37)	81 (47)	6361 (37)	79 (49)	456 (40)	423 (43)	343 (39)	535 (44)
Hispanic/others	5374 (31)	697 (30)	5338 (31)	35 (20)	5307 (31)	32 (20)	375 (33)	269 (27)	296 (33)	347 (28)
Non-Hispanic white	5600 (32)	582 (25)	5543 (32)	56 (33)	5495 (32)	49 (31)	300 (27)	296 (30)	247 (28)	350 (28)
Total	**17493**	**2322**	**17322**	**172**	**17163**	**160**	**1131**	**988**	**886**	**1232**

**Table 2:** Crude association between sociodemographic characteristics and alcohol-related harm.

	Family worries or complains about alcohol use	Alcohol use causing problems with others	Alcohol interfered with responsibilities	Drinking ever interfered with work/school/job/home	Drinking problem causing family/friend argues/problems
	OR (95% CI)	OR (95% CI)	OR (95% CI)	OR (95% CI)	OR (95% CI)
Sex					
Male	2.70 (2.47–2.96)	3.24 (2.35–4.47)	2.56 (1.86–3.53)	0.98 (0.82–1.17)	1.08 (0.90–1.30)
Female	1	1	1		
Age (group)					
≤34 years	2.05 (1.71–2.46)	2.57 (1.36–4.85)	5.63 (2.46–12.91)	2.03 (1.36–3.03)	1.23 (0.85–1.79)
35–49 years	2.75 (2.30–3.29)	2.33 (1.22–4.45)	4.15 (1.78–9.65)	2.07 (1.39–3.09)	1.75 (1.21–2.54)
50–64 years	2.19 (1.18–2.66)	2.12 (1.07–4.20)	1.77 (0.68–4.56)	1.91 (1.25–2.91)	1.84 (1.23–2.75)
≥65 years	1	1	1	1	1
Education (years)					
0–11	2.24 (1.96–2.58)	1.82 (1.15–2.87)	0.72 (0.44–1.17)	1.06 (0.81–1.39)	1.38 (1.05–1.81)
12	1.71 (1.50–1.96)	1.30 (0.83–2.03)	0.73 (0.48–1.13)	1.06 (0.82–1.39)	1.29 (0.99–1.68)
13–15	1.66 (1.45–1.91)	1.36 (0.86–2.14)	1.07 (0.72–1.60)	1.05 (0.80–1.37)	1.34 (1.02–1.76)
≥16	1	1	1	1	1
Income					
<$20,000	1.37 (1.22–1.53)	1.12 (0.75–1.68)	1.05 (0.70–1.55)	0.86 (0.70–1.07)	0.95 (0.77–1.18)
$20,000–29,999	1.17 (1.01–1.35)	1.39 (0.86–2.24)	0.77 (0.44–1.37)	1.05 (0.80–1.39)	0.85 (0.64–1.13)
$30,000–49,999	1.19 (1.04–1.35)	1.16 (0.75–1.80)	1.12 (0.73–1.71)	1.06 (0.84–1.34)	0.85 (0.67–1.08)
≥$50,000	1				
Ethnicity					
Non-Hispanic black	0.81 (0.73–090)	0.52 (0.35–0.78)	0.49 (0.32–0.73)	0.77 (0.63–0.95)	0.75 (0.61–0.92)
Hispanic/others	0.65 (0.58–0.72)	0.80 (0.57–1.13)	0.72 (0.50–1.03)	1.06 (0.86–1.31)	0.91 (0.73–1.12)
Non-Hispanic white	1	1	1	1	1
Physical health rating					
Good	1.34 (1.16–1.54)	1.02 (0.62–1.67)	1.45 (0.92–2.29)	1.24 (0.94–1.64)	1.22 (0.92–1.61)
Fair/poor	2.02 (1.75–2.33)	1.60 (0.98–2.62)	0.97 (0.53–1.76)	1.05 (0.79–1.39)	1.06 (0.80–1.40)
Very good/excellent	1	1	1	1	1
Mental health rating					
Good	1.59 (1.39–1.82)	1.26 (0.77–2.06)	1.01 (0.61–1.67)	1.01 (0.77–1.32)	1.08 (0.82–1.41)
Fair/poor	2.63 (2.24–3.08)	2.56 (1.51–4.36)	1.60 (0.87–2.94)	0.97 (0.72–1.32)	1.25 (0.92–1.70)
Very good/excellent	1	1	1	1	1
Smoke					
Yes	1.51 (1.19–1.92)	1.70 (0.66–4.37)	1.39 (0.52–3.68)	0.77 (0.50–1.17)	0.77 (0.50–1.18)
No	1	1	1	1	1

**Table 3:** Association between alcohol consumption measured as number of drinks per occasion and alcohol-related harm.

	Family worries or complains about alcohol use	Alcohol use causing problems with others	Alcohol interfered with responsibilities	Drinking ever interfered with work/school/job/home	Drinking problem causing family/friend argues/problems
	OR (95% CI)	OR (95% CI)	OR (95% CI)	OR (95% CI)	OR (95% CI)
**Model 1: crude association**					
Drinks per occasion					
≤2 drinks/occasion	1	1	1	1	1
2–4 drinks/occasion	1.99 (1.70–2.35)	2.32 (1.44–3.76)	2.58 (1.54–4.31)	1.26 (0.95–1.66)	1.32 (1.00–1.73)
≥5 drinks/occasion	4.38 (3.73–5.14)	3.27 (1.97–5.42)	3.28 (1.88–5.73)	1.69 (1.30–2.19)	2.09 (1.61–2.72)
**Model 2: adjusted association**					
Drinks per occasion					
≤2 drinks/occasion	1	1	1	1	1
2–4 drinks/occasion	1.40 (0.98–2.00)	1.88 (1.02–3.46)	1.98 (1.19–3.29)	1.17 (0.90–1.53)	1.26 (0.97–1.63)
≥5 drinks/occasion	3.33 (2.10–5.30)	2.43 (1.16–5.08)	2.64 (1.41–4.96)	1.61 (1.21–2.13)	2.17 (1.62–2.90)
Sex			—	—	—
Male	1.79 (1.39–2.79)	2.22 (1.17–4.22)	1.59 (0.99–2.58)		
Female	1	1	1		
Age (group)					
≤34 years	1.70 (0.85–3.40)	5.96 (0.79–45.04)	**5.46 (2.09–7.70)**	**3.80 (1.81–7.98)**	**2.35 (1.25–4.42)**
35–49 years	**2.07 (1.06–4.01)**	3.42 (0.44–26.70)	**4.38 (3.73–5.14)**	**3.45 (1.64–7.27)**	**3.80 (2.02–7.17)**
50–64 years	1.84 (0.92–3.69)	4.54 (0.57–36.35)	1.16 (0.92–3.02)	**3.20 (1.48–6.95)**	**3.53 (1.81–6.88)**
≥65 years	1	1	1	1	1
Education (years)			—	—	
0–11	1.02 (0.55–1.86)	1.73 (0.71–4.24)			**1.55 (1.08–2.22)**
12	0.68 (0.38–1.21)	1.11 (0.48–2.60)			1.21 (0.86–1.71)
13–15	0.82 (0.44–1.51)	1.16 (0.49–2.72)			1.35 (0.96–1.89)
≥16	1	1			1
Income			—	—	—
<$20,000	1.57 (0.91–2.69)	0.92 (0.42–1.99)			
$20,000–29,999	**2.19 (1.23–3.90)**	1.16 (0.48–2.80)			
$30,000–49,999	**1.74 (1.04–2.98)**	1.33 (0.65–2.73)			
≥$50,000	1	1			
Ethnicity					
Non-Hispanic black	0.58 (0.22–1.54)	**0.40 (0.17–0.95)**	**0.50 (0.29–0.88)**	**0.76 (0.58–0.99)**	**0.73 (0.57–0.99)**
Hispanic/others	1.29 (0.55–3.04)	0.59 (0.26–1.30)	**0.50 (0.29–0.85)**	1.09 (0.83–1.43)	0.93 (0.70–1.23)
Non-Hispanic white	1	1	1	1	1
Physical health rating		—	—	—	—
Good	1.31 (0.89–1.94)				
Fair/poor	1.46 (0.92–2.31)				
Very good/excellent	1				
Mental health rating			—	—	—
Good	**1.50 (1.02–2.20)**	1.39 (0.74–2.61)			
Fair/poor	**2.54 (1.52–4.25)**	**2.70 (1.24**–**5.84)**			
Very good/excellent	1	1			
Smoke		—	—	—	—
Yes	1.17 (0.80–1.73)				
No	1				

## References

[1] Zamboanga BL, Olthuis JV, Horton NJ, McCollum EC, Lee JJ, Shaw R (2009). Where’s the house party? Hazardous drinking behaviors and related risk factors. *Journal of Psychology: Interdisciplinary and Applied*.

[2] World Health Organization Global health risks: mortality and burden of disease attributable to selected major risks. http://www.who.int/healthinfo/global_burden_disease/GlobalHealthRisks_report_Front.pdf.

[3] Rehm J, Mathers C, Popova S, Thavorncharoensap M, Teerawattananon Y, Patra J (2009). Global burden of disease and injury and economic cost attributable to alcohol use and alcohol-use disorders. *The Lancet*.

[4] Rehm J, Room R, Monteiro M, Ezzati M, Lopez A, Rodgers A, Murray CJL (2004). Alcohol use. *Comparative Quantification of Health Risks: Global and Regional Burden of Disease Attributable to Selected Major Risk Factors*.

[5] Room R, Babor T, Rehm J (2005). Alcohol and public health. *The Lancet*.

[6] World Health Organization (2011). *Global Status Report on Alcohol and Health 2011*.

[7] Dawson DA, Li TK, Grant BF (2008). A prospective study of risk drinking: at risk for what?. *Drug and Alcohol Dependence*.

[8] Babor TF, Caetano R, Casswell S (2010). *Alcohol: No Ordinary Commodity: Research and Public Policy*.

[9] Andréasson S, Allebeck P (2005). Alcohol and health. A knowledge survey of alcohol’s positive and negative effects on our health. *Report R*.

[10] Naimi TS, Nelson DE, Brewer RD (2010). The intensity of binge alcohol consumption among U.S. adults. *The American Journal of Preventive Medicine*.

[11] Carlfjord S, Johansson K (2012). Association between frequency of heavy episodic drinking and self-reported consequences: a cross-sectional study in a Swedish population. *Alcohol and Alcoholism*.

[12] Measham F, Brain K (2005). ‘Binge’ drinking, British alcohol policy and the new culture of intoxication. *Crime, Media, Culture*.

[13] Fillmore MT, Jude R (2011). Defining “binge” drinking as five drinks per occasion or drinking to a.08% BAC: which is more sensitive to risk?. *The American Journal on Addictions*.

[14] Harding R, Stockley CS (2007). Communicating through government agencies. *Annals of Epidemiology*.

[15] Rehm JT, Room R, Taylor B (2008). Method for moderation: measuring lifetime risk of alcohol-attributable mortality as a basis for drinking guidelines. *International Journal of Methods in Psychiatric Research*.

[16] NIAAA (2004). *NIAAA Council Approves Definition of Binge Drinking*.

[17] Room R (2000). Measuring drinking patterns: the experience of the last half century. *Journal of Substance Abuse*.

[18] Caetano R (1997). The epidemiology of alcohol-related problems in the U.S.: concepts, patterns and opportunities for research. *Drugs and Society*.

[19] Cahalan D (1970). *Problem Drinkers*.

[20] Graham K, Bernards S, Knibbe R (2011). Alcohol-related negative consequences among drinkers around the world. *Addiction*.

[21] Babor TF, de la Fuente JR, Saunders J, Grant M (1989). *AUDIT: The Alcohol Use Disorders Identification Test: Guidelines for Use in Primary Health Care*.

[22] Room R Spouse reports versus self reports of drinking in general population surveys.

[23] Cahalan D, Room R (1974). *Problem Drinking among American Men*.

[24] Mäkelä P, Paljärvi T (2008). Do consequences of a given pattern of drinking vary by socioeconomic status? A mortality and hospitalisation follow-up for alcohol-related causes of the Finnish drinking habits surveys. *Journal of Epidemiology and Community Health*.

[25] Mäkelä P, Keskimäki IT, Koskinen S (2003). What underlies the high alcohol related mortality of the disadvantaged: high morbidity or poor survival?. *Journal of Epidemiology and Community Health*.

[26] Hawkins JD, Graham JW, Maguin E, Abbott R, Hill KG, Catalano RF (1997). Exploring the effects of age of alcohol use initiation and psychosocial risk factors on subsequent alcohol misuse. *Journal of Studies on Alcohol*.

[27] Nolen-Hoeksema S (2004). Gender differences in risk factors and consequences for alcohol use and problems. *Clinical Psychology Review*.

[28] Diehl A, Croissant B, Batra A, Mundle G, Nakovics H, Mann K (2007). Alcoholism in women: is it different in onset and outcome compared to men?. *European Archives of Psychiatry and Clinical Neuroscience*.

[29] Carlson SR, Iacono WG, McGue M (2002). P300 amplitude in adolescent twins discordant and concordant for alcohol use disorders. *Biological Psychology*.

[30] Kessler RC, Crum RM, Warner LA, Nelson CB, Schulenberg J, Anthony JC (1997). Lifetime co-occurrence of DSM-III-R alcohol abuse and dependence with other psychiatric disorders in the national comorbidity survey. *Archives of General Psychiatry*.

[31] Stinson FS, Grant BF, Dawson DA, Ruan WJ, Huang B, Saha T (2005). Comorbidity between DSM-IV alcohol and specific drug use disorders in the United States: results from the national epidemiologic survey on alcohol and related conditions. *Drug and Alcohol Dependence*.

[32] Surís JC, Michaud PA, Akre C, Sawyer SM (2008). Health risk behaviors in adolescents with chronic conditions. *Pediatrics*.

[33] Plant M, Miller P, Thornton C, Plant M, Bloomfield K (2000). Life stage, alcohol consumption patterns, alcohol-related consequences, and gender. *Substance Abuse*.

[34] Wilsnack RW, Vogeltanz ND, Wilsnack SC (2000). Gender differences in alcohol consumption and adverse drinking consequences: cross-cultural patterns. *Addiction*.

[35] Mulia N, Ye Y, Greenfield TK, Zemore SE (2009). Disparities in alcohol-related problems among white, black, and hispanic Americans. *Alcoholism: Clinical and Experimental Research*.

[36] Copello AG, Velleman RDB, Templeton LJ (2005). Family interventions in the treatment of alcohol and drug problems. *Drug and Alcohol Review*.

[37] Bobak M, Room R, Pikhart H (2004). Contribution of drinking patterns to differences in rates of alcohol related problems between three urban populations. *Journal of Epidemiology and Community Health*.

[38] Kessler RC, Ustün TB (2004). The world mental health (WMH) survey initiative version of the world health organization (WHO) composite international diagnostic interview (CIDI). *International Journal of Methods in Psychiatric Research*.

[39] Kessler RC, Abelson J, Demler O (2004). Clinical calibration of DSM-IV diagnoses in the world mental health (WMH) version of the world health organization (WHO) composite international diagnostic interview (WMH-CIDI). *International Journal of Methods in Psychiatric Research*.

[40] World Health Organization (1991). *International Classification of Diseases (ICD-10)*.

[41] American Psychiatric Association (1994). *Diagnostic and Statistical Manual of Mental Disorders (DSMIV)*.

[42] Hasin DS, Stinson FS, Ogburn E, Grant BF (2007). Prevalence, correlates, disability, and comorbidity of DSM-IV alcohol abuse and dependence in the United States: results from the national epidemiologic survey on alcohol and related conditions. *Archives of General Psychiatry*.

[43] Degenhardt L, Chiu WT, Sampson N, Kessler RC, Anthony JC (2007). Epidemiological patterns of extra-medical drug use in the United States: evidence from the National Comorbidity Survey Replication, 2001–2003. *Drug and Alcohol Dependence*.

[44] Grant BF, Hasin DS, Chou SP, Stinson FS, Dawson DA (2004). Nicotine dependence and psychiatric disorders in the United States: results from the national epidemiologic survey on alcohol and related conditions. *Archives of General Psychiatry*.

[45] Kessler RC, Wai TC, Demler O, Walters EE (2005). Prevalence, severity, and comorbidity of 12-month DSM-IV disorders in the national comorbidity survey replication. *Archives of General Psychiatry*.

[46] Greenland S, Lash TL, Rothman KJ (2008). Bias analysis. *Modern Epidemiology*.

[47] Studenmund AH, Cassidy HJ (1987). *Using Econometrics: A Practical Guide*.

[48] Harford TC, Grant BF, Yi HY, Chen CM (2005). Patterns of DSM-IV alcohol abuse and dependence criteria among adolescents and adults: results from the 2001 national household survey on drug abuse. *Alcoholism: Clinical and Experimental Research*.

[49] Álvarez FJ, Fierro I, del Río MC (2006). Alcohol-related social consequences in Castille and Leon, Spain. *Alcoholism: Clinical and Experimental Research*.

[50] Rehm J, Gmel G (1999). Patterns of alcohol consumption and social consequences. Results from an 8-year follow-up study in Switzerland. *Addiction*.

[51] Gmel G, Rehm J, Room R, Greenfield TK (2000). Dimensions of alcohol-related social and health consequences in survey research. *Journal of Substance Abuse*.

[52] Rossow I, Romelsjö A (2006). The extent of the “prevention paradox” in alcohol problems as a function of population drinking patterns. *Addiction*.

[53] Rehm J, Room R, Graham K, Monteiro M, Gmel G, Sempos CT (2003). The relationship of average volume of alcohol consumption and patterns of drinking to burden of disease: an overview. *Addiction*.

[54] Corrao G, Bagnardi V, Zambon A, La Vecchia C (2004). A meta-analysis of alcohol consumption and the risk of 15 diseases. *Preventive Medicine*.

[55] Miller JW, Naimi TS, Brewer RD, Jones SE (2007). Binge drinking and associated health risk behaviors among high school students. *Pediatrics*.

[56] Pillai A, Nayak MB, Greenfield TK, Bond JC, Nadkarni A, Patel V (2013). Patterns of alcohol use, their correlates, and impact in male drinkers: a population-based survey from Goa, India. *Social Psychiatry and Psychiatric Epidemiology*.

[57] Kullgren G, Alibusa S, Birabwa-Oketcho H (2009). Problem drinking among patients attending primary healthcare units in Kampala, Uganda. *African Journal of Psychiatry*.

[58] Obot I, Room R (2005). *Alcohol, Gender and Drinking Problems: Perspectives from Low and Middle Income Countries*.

[59] Wilkinson R, Marmot M (2003). *Social Determinants of Health: The Solid Facts*.

[60] Room R, Mäkelä K (2000). Typologies of the cultural position of drinking. *Journal of Studies on Alcohol*.

[61] Knibbe RA, Joosten J, Choquet M, Derickx M, Morin D, Monshouwer K (2007). Culture as an explanation for substance-related problems: a cross-national study among French and Dutch adolescents. *Social Science and Medicine*.

[62] Johnson FW, Gruenewald PJ, Treno AJ, Taff GA (1998). Drinking over the life course within gender and ethnic groups: a hyperparametric analysis. *Journal of Studies on Alcohol*.

[63] Caetano R, Clark CL (1999). Trends in situational norms and attitudes toward drinking among whites, blacks, and hispanics: 1984–1995. *Drug and Alcohol Dependence*.

[64] National Institute on Alcohol Abuse and Alcoholism http://pubs.niaaa.nih.gov/publications/AA70/AA70.htm.

[65] Timko C, Sutkowi A, Pavao J, Kimerling R (2008). Women’s childhood and adult adverse experiences, mental health, and binge drinking: the California women’s health survey. *Substance Abuse: Treatment, Prevention, and Policy*.

[66] Okoro CA, Brewer RD, Naimi TS, Moriarty DG, Giles WH, Mokdad AH (2004). Binge drinking and health-related quality of life: do popular perceptions match reality?. *The American Journal of Preventive Medicine*.

